# Characterization of a Sequential UV Photolysis-Biodegradation Process for Treatment of Decabrominated Diphenyl Ethers in Sorbent/Water Systems

**DOI:** 10.3390/microorganisms8050633

**Published:** 2020-04-27

**Authors:** Yi-Tang Chang, Wei-Liang Chao, Hsin-Yu Chen, Hui Li, Stephen A. Boyd

**Affiliations:** 1 Department of Microbiology, Soochow University, Shilin District, Taipei 11102, Taiwan; wlchao@scu.edu.tw (W.-L.C.); fish3265175@gmail.com (H.-Y.C.); 2 Department of Plant, Soil and Microbial Science, Michigan State University, East Lansing, MI 48824, USA; lihui@msu.edu

**Keywords:** decabrominated diphenyl ether, emerging contaminant, sequential photolysis- microbial biodegradation, PBDE congeners, *Achromobacter* spp.

## Abstract

Decabrominated diphenyl ether (BDE-209) is a primary component of the brominated flame retardants used in a variety of industrial and domestic applications. BDE-209 bioaccumulates in aquatic organisms and has been identified as an emerging contaminant that threatens human and ecosystem health. Sequential photolysis-microbial biodegradation processes were utilized here to treat BDE-209 in clay- or soil-water slurries. The removal efficiency of BDE-209 in the clay-water slurries was high; i.e., 96.5%, while that in the soil-water slurries was minimal. In the clay-water slurries the first order rate constants for the UV photolysis and biodegradation of BDE-209 were 0.017 1/day and 0.026 1/day, respectively. UV wavelength and intensity strongly influenced the BDE-209 photolysis and the subsequent biodegradation of photolytic products. Facultative chemotrophic bacteria, including *Acidovorax* spp., *Pseudomonas* spp., *Novosphingobium* spp. and *Sphingomonas* spp., were the dominant members of the bacterial community (about 71%) at the beginning of the biodegradation; many of these organisms have previously been shown to biodegrade BDE-209 and other polybrominated diphenyl ether (PBDE) congeners. The *Achromobacter* sp. that were isolated (NH-2; NH-4; NH-6) were especially effective during the BDE-209 degradation. These results indicated the effectiveness of the sequential UV photolysis and biodegradation for treating certain BDE-209-contaminated solids; e.g., clays; in bioreactors containing such solids as aqueous slurries. Achieving a similar treatment effectiveness for more heterogeneous solids containing natural organic matter, e.g., surface solids, appears to be significantly more difficult. Further investigations are needed in order to understand the great difference between the clay-water or soil-water slurries.

## 1. Introduction

Polybrominated diphenyl ethers (PBDEs), especially the penta-, octa- and deca- brominated congeners, constitute a major class of the commercial brominated flame retardants (BFR). These BFRs are used in plastics, textiles and in wire insulation, and hence are found in many commercial products such as automobiles. Decabrominated diphenyl ether (BDE-209) has continued to be used as a BFR even after the global prohibition of penta-BDE and octa-BDE, although its use has been restricted in the EU and the USA under the United Nations Stockholm Convention (http://www.pops.int/). Large quantities of solid waste containing BDE-209, in particular electronic or electrical wastes (e-waste), continues to be discarded inappropriately resulting in the release of BDE-209 into the environment [1]. As a result, PBDEs are commonly found in aquatic sediments, wastewater and industrial sludges, as well as soils. BDE-209 is the most dominant congener of PBDEs present in contaminated soil/sediment samples collected throughout the world. The percentage contribution of BDE-209 to the total PBDE contamination in soil has often been reported to be greater than 60% and may exceed 90%. For example, one detailed investigation of sediments from the Laurentian Great Lakes (GL) showed that BDE-209 was the dominant congener, accounting for more than 90% of the total mass of the five major PBDE congeners present [2]. The accumulated BDE-209 in GL sediments has adversely impacted the GL ecosystem; this is an ongoing problem since BDE-209 is still present in many commercial/industrial products. Thus, BDE-209 remains an important and continuing source of PBDEs in the GL region.

PBDEs have been identified as emerging contaminants (EC), which are defined as chemicals that have been detected in the environment that may have ecological or human health impacts but are not sufficiently regulated under current environmental law. Many PBDEs congeners, such as tetra-BDEs to hepta-BDEs, have been described as both persistent organic pollutants (POPs) and endocrine disrupting chemicals (EDCs). Environmental scientists have begun to pay attention to the chronic toxicity of these chemicals because they typically have long half-lives in the environment. The human health effects of BDE-209 have been shown to involve the liver, heart, kidneys and the thyroid, and to include various reproductive, developmental and neurobehavioral effects. For example, using Sprague-Dawley rats as a mammalian model, BDE-209 has been shown to induce oxidative stress and inflammation, leading to endothelial dysfunction and morphological/ultrastructural damage to the heart and the abdominal aorta [3]. Human embryonic kidney cells, when treated with BDE-209, show alterations to nucleosome organization via significant changes in the expression of various histone gene clusters [4]. Based on the aforementioned studies, it has become necessary to continuously monitor the levels of BDE-209 in soils and sediments, and to develop aggressive strategies to effectively remediate BDE-209-contaminated soils and sediments.

BDE-209 photodegradation occurs when free-radicals are formed by UVA and UVB radiation which then attacks the bromines present in PBDEs. UV photolysis to remove PBDEs has been applied to liquid matrices including organic solvents and water, and to gas and solid phases including TiO_2_ [5]. UV photolysis of BDE-209 has also been shown to occur in a variety of soils and soil components of differing particle sizes including whole natural soil, sand and clay (montmorillonite and kaolinite) as well as sediments [6]. The UV light energy used in prior studies to irradiate soil contaminated with PBDEs originated from a variety of sources, each with characteristic wavelengths [6,7]. These include natural sunlight, an artificial sunlight lamp (250–305 nm), a black-light lamp (305–440 nm) and a mercury UV-lamp (300–400 nm). However, little information was available on the BDE-209 photolysis resulting from the UV irradiation of a soil slurry, i.e., a soil saturated with water, despite the fact that this type of environment forms a major part of aquatic ecosystems throughout the world.

A sequential treatment process involving both UV radiation and biodegradation has demonstrated potential as a technique for POP/EDC removal. Prior studies have shown a variety of UV-biological treatment processes that have been used to remove target POPs/EDCs (e.g., polycyclic aromatic hydrocarbons (PAHs), chlorphenols and pesticides) in various aqueous solutions and organic solvents of differing compositions (Table S1). This approach to the removal of POPs/EDCs was in general more effective compared to any single treatment involving UV photolysis or aerobic/anaerobic biodegradation. An advantage of a sequential treatment approach to POP/EDC biodegradation is that the effectiveness of biodegradation is increased by prior UV photolysis pretreatment. The generation of free radicals in a bioreactor can be easily controlled by using different energy intensities associated with various UV irradiation devices. Although an economical green remediation technology for POP/EDC-contaminated liquids is likely to be provided by a sequential UV-biological treatment process, little information is available on the use of such a sequential treatment for POP/EDC removal from solids in a water-saturated system such as a soil slurry.

Our aim in this study was to evaluate a sequential photolysis-bioremediation process for the treatment of BDE-209-contaminated natural solids in an aqueous slurry system. Such a system avoided the need to dewater, and allowed for the optimal mixing and control of redox conditions. Importantly, this type of system is ubiquitous in aquatic ecosystems (e.g., wetlands, river/lake/ocean sediments, water reservoirs) and agricultural farms (e.g., paddy field, aquafarm), which are common sinks for the accumulation of POPs/EDCs [1,2]. Moreover, soil slurry reactors (SSRs) are central to many important *ad-situ* or *ex-situ* remediation technologies [8]. Treatment in SSRs has become one of the best options for the bioremediation of soils polluted by POPs/EDCs under controlled environmental conditions. SSRs are often used to determine the actual potential of a biological strategy in the final restoration of a contaminated soil. The ability to remove BDE-209 by the bacterial mixed culture was confirmed when BDE-209 was used as the sole carbon source in soil/water slurry systems [9,10]. Because bioremediation is often slow or incomplete, it is important and necessary to develop more effective strategies such as a sequential UV-biological treatment process for POPs/EDCs. Hence, a SSR was designed that incorporated UV photolysis as a pretreatment and aerobic biodegradation as a second sequential process. The removal efficiency and the kinetics of the BDE-209 degradation for each process were assessed for two sorbent/water systems. We assessed the effectiveness of the treatment in a comparatively pure clay (montmorillonite) vs. a more complex whole natural soil which contained both organic matter and mineral particles of differing sizes and compositions. The effects of the UV irradiation wavelength and intensity on the degradation of BDE-209 in the sorbent/water systems were assessed. Changes in the bacterial community during BDE-209 biodegradation were monitored by high throughput sequencing technology. Finally, the bacterial strains were isolated from the SSR, identified and evaluated for their ability to utilize BDE-209 as a sole carbon source. These isolated strains can then be used as potential candidates for the bioremediation of PBDEs or POP/EDC-contaminated slurries in the future.

## 2. Materials and Methods

### 2.1. Chemicals and Bacterial Mixed Cultures

The BDE-209 (Alfa Aesar, Karlsruhe, Germany, 99% purity) used in this study had a purity above 99%. A standard solution of BDE-209 (Cambridge Isotope Laboratories, Andover, MA, USA, 50 mg/L) was made by dissolving BDE-209 in hexane and this was used when required for the analysis of the concentration of BDE-209. All organic solvents were HPLC grade and had a purity of greater than 99.9%. All the other chemicals used in this study were reagent grade and had a purity of greater than 99%. The Milli-Q water was double-distilled and deionized by a Millipore water purification system.

A bacterial mixed culture capable of BDE-209 biodegradation was previously obtained by sampling activate sludge that contained 256.8 BDE-209 ng/g; this was obtained from a biological treatment plant at Nei-Hu Wastewater Treatment Plant (WWTP, Taipei, Taiwan). The details of the enrichment process used on the Nei-Hu bacterial mixed culture was outlined in our previous research [9]. The ability to remove BDE-209 by the Nei-Hu bacterial mixed culture was shown to be 70% biodegradation of 25 mg/kg BDE-209 over 6 months when BDE-209 was the sole carbon source in a soil/water slurry system [10].

### 2.2. BDE-209-Contaminated Sorbent/Water Systems

Two types of sorbents were selected to be used as a sorbent/water system. The physical-chemical characteristics of these sorbents are shown in Table S2. A whole natural soil was collected from Taichung City, Taiwan. The soil sample was air-dried then sieved to obtain particles of less than 2.0 mm; finally, it was then stored at room temperature until the experiments commenced. In order to prepare a 20 μg/g BDE-209 sorbent/water system using the soil, 2.70 mg BDE-209 was dissolved in acetone and this was then completely mixed with 135.0 g soil, then 3365 mL of Milli-Q water was added and mixed with the soil after the acetone evaporated completely. The soil/water ratio was designed to be 1.0 g soil to 25.0 1/mL. The second sorbent used in this study was pure Ca-montmorillonite and this was purchased from the Clay Minerals Society, Purdue University, USA. For the clay/water slurry system, 5.20 mg BDE-209 was dissolved in acetone and this was completely mixed with 259.0 g clay and 3240 mL Milli-Q water in the same manner as described above. The clay/water ratio soil was designed to be 2.0 g of clay to 25.0 1/mL.

### 2.3. A Sequential Treatment Process Involving UV Photolysis Followed by Biodegradation

#### 2.3.1. The SSR Design

**Figure 1:**
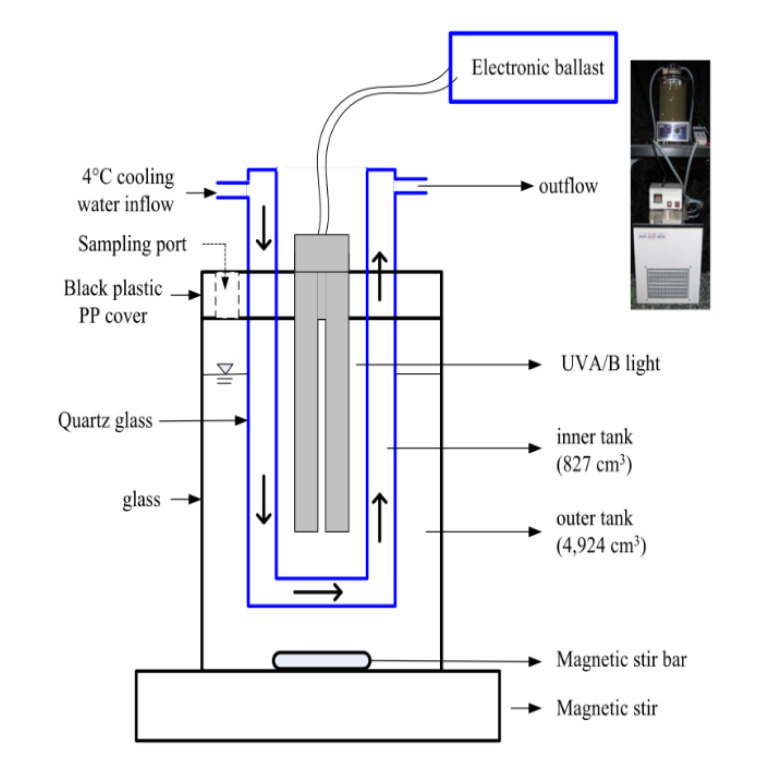
The soil slurry reactor (SSR) used in this study.

The detailed design of the SSR that was used for the sequential treatment of BDE-209 by the UV photolysis and biodegradation is shown in Figure 1. The SSR consisted of two reactors constructed from different materials, namely an outer glass reactor and an inner quartz tank. The volume of the inner and outer tanks were 4924 cm^3^ and 827 cm^3^, respectively. A polypropylene plastic board was used to cover the top of the bioreactor; this meant that there was no exposure to UV irradiation from the external environment, only from the UV lamp inside. The UV lamps used in this study were a 365-nm UVA lamp (18WPL-L/10 Philips, Poland) and a 312-nm UVB lamp (13WPL-L/Philips, Poland); these were setup as alternatives in the middle of the quartz inner tank. UV light is able to easily penetrate the quartz into the sorbent/water system and this radiation is able to generate significant free radicals; these will then be able to chemically alter then cleave the structure of BDE-209 and its metabolites. The intensity of UV irradiation on the outer surface of the inner quartz tank was measured and was found to range from 1.64 to 2.71 mw/cm^2^ when the 365-nm UVA lamp was used and from 0.58 to 0.97 mw/cm^2^ when the 312 nm UVB lamp was used. The sorbent/water systems, namely the clay/water slurry system and the soil/water slurry system, were added individually to the space between the two tanks. The complete mixing of both sorbent/water systems during the BDE-209 photolysis/biodegradation was brought about by the use of a magnetic stir plate set at 300 rpm. Since the heat from the UV irradiation system was continuously entering the SSR, cooling via a circulating system was set up in order to keep the temperature of the system at a steady 25 °C during all the experiments.

#### 2.3.2. Degradation of BDE-209 in the SSR by Sequential Treatment

The designed sequential treatment process for the BDE-209 degradation using the two different sorbent/water systems was carried out in the SSR described above. The first treatment process involved UV photolysis to degrade BDE-209 in a sorbent/water system for 35 days. An absolutely dark environment (0 mw/cm^2^) was set up in order to avoid a reductive debromination by outside radiation or by various other chemical reactions. The samples were collected on the first, seventh, 14th, 21st, 28th and 35th days. A constant sorbent-to-water ratio was maintained in the SSR in order to prevent the sampling procedure from having an effect on the BDE-209 degradation. After the UV photolysis, the BDE-209 biodegradation was carried out in the same bioreactor system and this involved a number of steps. Specifically, over a total of eight days (from the 35th day to the 42nd day), the UV-pretreated sorbent/water samples were removed from the photolysis process and set up as a biodegradation process using the same bioreactor. The change consisted of three major processes. Firstly, the aqueous solution present in the sorbent/water bioreactor was discarded. Secondly, the sorbents were autoclaved at least twice to remove any bacteria that had grown in the reactor during the UV photolysis. Thirdly, a mineral salt basal (MSB) medium [9] was mixed uniformly with the sterilized soil in order to allow for biodegradation to take place in an aquatic environment; the sorbent/water ratio during the biodegradation was the same as during the UV photolysis. The above processes did result in a loss of BDE-209 and the concentration was found to have decreased from 9.98 ± 1.80 ng/g (day 35) to 7.72 ± 2.01 ng/g (day 42). Then, an inoculum from the Nei-Hu WWTP was sub-cultured to create a microbial inoculating culture capable of BDE-209 biodegradation and this was added to the SSR to give 1×10^5^ CFU/mL of mixed bacterial culture when grown on R2A agar [9]. The reactor was then incubated with 300 rpm stirring, which allowed for the complete mixing at room temperature (25 °C on average) for the experimental period. The SSR was maintained at 2.0 dissolved oxygen mg/L or above in an absolutely dark environment (0 mw/cm^2^); the experiment continued from day 43 to day 140 (98 days). The sorbent/water system was sampled regularly on the 43rd, 74th, 105th and 140th day to allow for analysis. A constant sorbent-to-water ratio was maintained in order to prevent the sampling procedure from affecting the BDE-209 biodegradation. The control sorbent/water systems were used, the first stage of which consisted of biodegradation alone in an absolutely dark environment (no UV irradiation), while the second stage consisted of sterilized BDE-209-MSB that had been poisoned with 32.5g HgCl_2_. These were analyzed in a similar manner to that of the non-control systems.

### 2.4. BDE-209 and PBDEs Analysis

An ultrasound extraction method was selected as an appropriate approach to be used for the analysis of BDE-209. For the GC analysis, a solid sample was obtained from the sorbent/water system by high-speed centrifugation in a polytetrafluoroethylene centrifuge tube. Precisely 1.000 g of the solid sample was then extracted using 10.0 mL of solvent, namely a mixture of hexane and acetone (1:1, *v/v*). The solid sample was extracted twice for 30 min using an ultrasonic water bath (output energy of 200 W at a frequency of 40 kH) at room temperature. The water level in the bath was adjusted to be equal to that of the extraction solvent in the PTFE centrifuge tube. For the GC/MS analysis, purer extracts were required and an extra cleaning process was introduced that involved a 16 × 300 mm glass chromatography column packed with self-prepared 60–200 mesh acidic silica gel and specific glass wool. The sample extract was eluted from the acidic silica gel column using 20 mL hexane and then concentrated to near dryness by rotary evaporation. Finally, the residue was dissolved in 1.0 mL hexane in a flask and filtered through a 0.22 μm PTFE syringe filter. A final volume of 1 μL was injected during the chromatographic analysis. The stock solutions of BDE-209 were prepared by serial dilution in hexane and these were stored until use in an individual dark-brown glass container at 4 °C to prevent BDE-209 photolysis.

The concentration of BDE-209 was measured by GC (HP 5890 Series II) using a pulsed discharge electron capture detector (PDECD, Valco Models D-2-I). The conditions used for the BDE-209 analysis were as follows: a Restek^®^ Rxi-5HT column was selected for the separation based on the literature. The carrier gas consisted of a mixture containing 95% (*v/v*) helium (99.999% purity) and 5% (*v/v*) refined methane at a high purity and the carrier gas ran at a constant flow rate of 10 mL/min. The injection port and detector of the GC/PDECD were maintained at 320 °C and 380 °C, respectively. The temperature of the GC oven was initially set at 110 °C for 1 min and programmed so that the temperature increased at 40 °C/min until 200 °C was reached at which point it was held at 200 °C for 3 min; this was followed by a final ramp up to 330 °C at a rate of 10 °C/min. The temperature was then maintained at 330 °C for 4.75 min. The voltage of the PDECD was set at 30 V. Finally, 3 μL of the sample extract dissolved in hexane or 3 μL of the standard BDE-209 solution dissolved in hexane was injected into the GC/PDECD in the splitless mode. The method detection limit (MDL) for measuring BDE-209 in the sorbent/water systems was found to be 0.032 μg/g.

The 23 PBDE congeners were analyzed using a GC (HP 6890) equipped with an GC PAL autosampler (Unichrom Scientific Co. Ltd., Shanghai, China) that was coupled to an HSMS-700 high-performance double focusing magnetic sector mass spectrometer (JEOL Ltd., Tokyo, Japan). An Agilent^®^ DB-5HT capillary column was used for the separation of the PBDE congeners. The carrier gas was helium with a purity of 99.999% and was delivered at a rate of 1 mL/min. The GC oven temperature was programmed to increase in temperature from 110 °C (5 m) to 200 °C (5.5 m) at 40 °C 1/min then to increase to 330 °C at 10 °C 1/min; this temperature was then held for 2.25 m. The injector temperature was set at 320 °C and the separation was carried out in the splitless mode. The sample consisted of 1 mL of either the sample extract or the standard solution, both in hexane. To condition the MS, electron ionization (EI) was performed at 38 eV. The electron multiplier voltage and emission current were set up at 10 kV and 500 mA, respectively. The source temperature of the MS was set at 320 °C. The resolving power of the analyzer was >10,000. The MS was operated in the selected ion monitoring (SIM) mode and used eight descriptors to analyze the 23 PBDE congeners.

### 2.5. Bacterial Community Analysis

Metagenome analysis has become one of the most powerful techniques available for the analysis of the bacterial communities present in different ecosystems. Illumina sequencing technology is a widely adopted next-generation sequencing (NGS) technology that has been responsible for generating more than 90% of the world’s sequencing data. This approach was applied to assess the changes in the bacterial communities during the BDE-209 biodegradation at different time points (day 43; day 105; day 140). DNA samples were extracted using a Soil Genomic DNA Purification Kit (GeneMark, Taiwan). The bacterial communities were analyzed using the 27F-519R primer set to amplify the V1–V3 domains of 16S rDNA on an Illumina 454 stage (Genomics, Taiwan) [11]. The DNA sequences present in the amplified samples were processed as an amplicon library and this was followed by sequencing using synthesis sequencing. Finally, after the results were subjected to cluster generation, the bacterial communities present in the samples during the BDE-209 biodegradation were explored using the website http://basespace.illumina.com/. The sequences found on the day 43, day 105 and day 140 samples, which were generated by the Illumina 454 sequencing platform, are shown in Table S3, Table S4 and Table S5, respectively.

### 2.6. Bacterial Strains Capable of Utilizing BDE-209 as a Sole Carbon Source in the Sorbent/Water System

The bacterial strains capable of using BDE-209 as a sole carbon source were isolated during the BDE-209 biodegradation in the sorbent/water system. To do this, 10 mL the sorbent/water mixture was sampled and diluted 10-times serially on days 43, 74, 105 and 140. A 0.2 μL inoculum was spread on the purified agar plates that contained 20 μg/L (below theoretical water solubility) BDE-209 as the sole carbon source plus MSB. To prevent bacterial strains from utilizing the organic residues present in agar as a carbon source, purified agar was obtained by washing the agar powder by Milli-Q water at least three times before medium preparation. Pure strains as single colonies were identified after incubation at 25 °C for 14 days. These isolated strains were confirmed as being able to grow well on the BDE-209-MSB agar plates by the streak method technique and this streaking was repeated at least 3–5 times. Finally, these strains were identified by the Mission Biotech company (Taiwan) based on the V1–V8 domain sequences of their 16s rDNA regions.

A Biolog MT2 microplate (Hayward, CA, USA)-based assay was selected for the rapid identification and evaluation of the ability of a given bacterial strain to utilize BDE-209 as a sole carbon substrate. Each Biolog MT2 microplate well was created as a sole carbon source substrate well (20 mg/L BDE-209) in MSB; in addition, an equal concentration of tetrazolium violet dye, which is sensitive to the oxidation of a carbon source and bacterial respiration, was added [12]. The microplate wells were then inoculated with 150 μL of each isolated bacterial strain, which gave a bacterial density or OD590 of between 0.2 and 0.4, 108 CFU/mL on R2A agar; the incubation was carried out at 25°C in the dark for 2 days. The controls for bacterial respiration were set up for each isolated strain using MSB-contained Biolog MT2 microplate wells that did not contain any carbon source. The OD value of each microplate well was measured using an ELISA Reader at 590 nm. A positive response, namely the ability to carry out BDE-209 biodegradation, was assessed using (Sample OD590–Control OD590) based on the purple color present in each of the Biolog MT2 wells. Three levels of BDE-209 utilization were defined using the relative OD590 values of the strains (see above) obtained; these were: strong (>0.3), medium (0.1–0.3), and weak (<0.1).

## 3. Results

### 3.1. BDE-209 Removal

**Figure 2:**
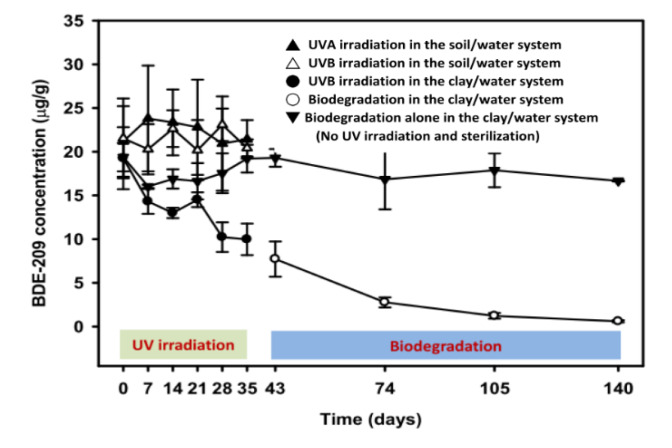
The concentration of decabrominated diphenyl ether (BDE-209) after the sequential treatment with photolysis and biodegradation in the SSR system.

The individual and combined effectiveness of the sequential process involving UV photolysis and biodegradation for the treatment of BDE-209 in the bioreactor containing clay- and soil-water slurries are shown in Figure 2. This study compared the two sorbent/water systems first for BDE-209 photolysis by either UVA/B irradiation. During the initial UV photolysis stage (0–35 days), the BDE-209 degradation by UV irradiation was different for the two sorbent/water systems. Using the soil/water slurry system, the concentration of BDE-209 stayed relatively constant, i.e., within the range 20.29 ± 2.87 μg/g to 23.80 ± 6.05 μg/g, during the irradiation with either UVA or UVB over a period of 35 days. The soil organic matter (SOM) affected the performance of the BDE-209 photolysis (see Section 4.1). In contrast the clay/water system during the irradiation with UVB-based photolysis was selected due to the shorter wavelength of UV having a higher photodegradation efficiency (see Section 4.2), and the decrease was significant (~50%). The photolysis process was stopped based on (1) a decrease in the concentration BDE-209 to 50% was achieved, and (2) a significant increase occurring in the concentration of low brominated PBDEs (Figure S3) and hydroxylated PBDEs (PBDE-OH). When the concentration of BDE-209 by UVB irradiation was successfully decreasing in the clay/water system, we skipped the UVA-based photolysis process and stepped into the next stage of biodegradation. Specifically, when using the UVB irradiation, BDE-209 decreased from 19.26 ± 3.55 μg/g to 9.98 ± 1.80 μg/g over the 35 days. The degradation of PBDE by photolysis (or biodegradation) followed the first-order kinetics, and the first-order rate constant and half-life of BDE-209 under the UVB photolysis were c. 0.017 1/day (*r^2^* = 0.92) and 33 days, respectively. Considering these results, the BDE-209 biodegradation following UVB photolysis using the clay/water slurry system was selected as the model multi-stage system. Using this system, the BDE-209 biodegradation commenced at concentration of 7.72 ± 2.01 μg/g following inoculation on the 43rd day, and continued to a final concentration of 0.60 ± 0.12 μg/g on the 140th day. The extent of the BDE-209 biodegradation was c. 92.23%, and the first-order rate constant and half-life of the BDE-209 biodegradation were c. 0.026 1/day (*r^2^* = 0.99) and 22 days, respectively. Thus, the total BDE-209 removed by the sequential UVB photolysis-biodegradation was c. 96.47% in the clay/water slurry system over a period of 140 days. In the sterile (HgCl_2_) control, the concentration of BDE-209 remained in the range of 16.0 ± 0.2 μg/g to 19.4 ± 1.6 μg/g during the 140-day treatment period.

### 3.2. Bacterial Community Analysis

**Figure 3:**
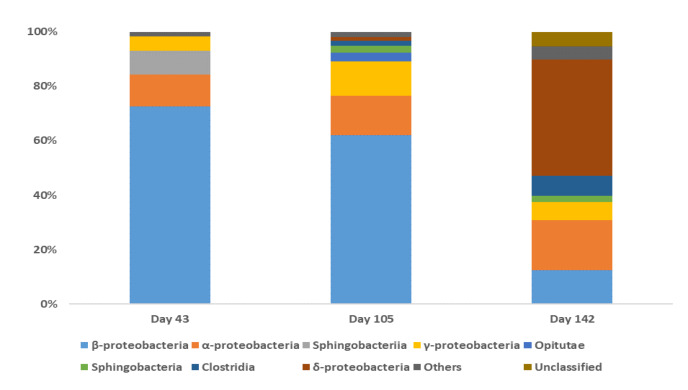
Community analysis of the class-level bacteria present during the BDE-209 biodegradation in the clay/water slurry system.

**Figure 4:**
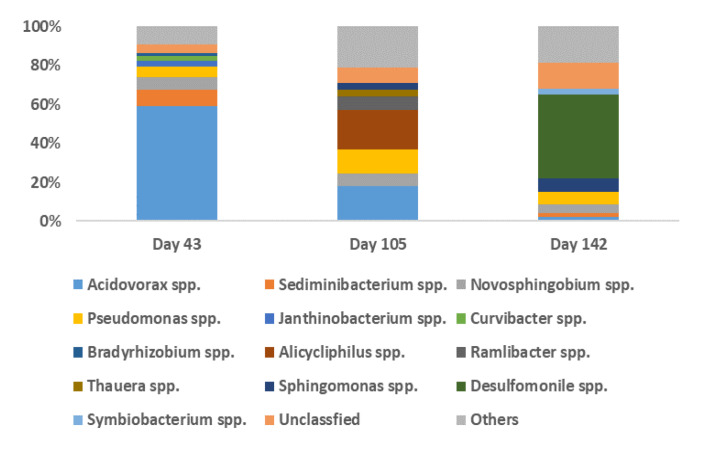
Community analysis of the genus-level bacteria present during the BDE-209 biodegradation in the clay/water slurry system.

The phyla Proteobacteria and Bacteroidetes appeared to play important roles in the biodegradation of BDE-209 in the clay/water slurry system accounting for 82.13% to 90.47% and 2.31% to 8.78% of the total bacterial population, respectively. The changes in the bacterial community that occurred during BDE-209 biodegradation based on a class level analysis are shown in Figure 3. The major classes (>1% of total) present in the bacterial community consisted of β-proteobacteria (72.83%), α-proteobacteria (11.47%), Sphingobacteriia (8.78%) and γ-proteobacteria (5.24%) on day 43, and β-proteobacteria (61.93%), α-proteobacteria (14.59%), γ-proteobacteria (12.67%), Opitutae (3.07%), Sphingobacteria (2.63%), Clostridia (1.81%) and δ-proteobacteria (1.28%) on day 105. At the end of the treatment period (day 140), the major classes (>2% of total) were δ-proteobacteria (43.80%), α-proteobacteria (18.94%), β-proteobacteria (12.66%), Clostridia (7.56%), γ-proteobacteria (6.73%) and Sphingobacteria (2.31%). Changes in the bacterial communities during the BDE-209 biodegradation based on the genus level analysis are shown in Figure 4. On day 43, the major species (>1%) present were *Acidovorax* spp. (58.88%), *Sediminibacterium* spp. (8.67%), *Novosphingobium* spp. (6.56%), *Pseudomonas* spp. (5.12%), *Janthinobacterium* spp. (3.02%), *Curvibacter* spp. (2.67%) and *Bradyrhizobium* spp. (1.48%). On day 105, the major species (>3%) present consisted of *Alicycliphilus* spp. (20.37%), *Acidovorax* spp. (17.89%), *Pseudomonas* spp. (12.07%), *Ramlibacter* spp. (7.13%), *Novosphingobium* spp. (6.72%), *Thauera* spp. (3.60%) and *Sphingomonas* spp. (3.04%). Finally, on day 140, the eight major species detected were *Desulfomonile* spp. (43.21%), *Sphingomonas* spp. (7.00%), *Pseudomonas* spp. (6.08%), *Novosphingobium* spp. (4.73%), *Symbiobacterium* spp. (2.98%), *Acidovorax* spp. (2.07%) and *Sediminibacterium* spp. (1.96%).

### 3.3. Isolated Bacterial Strains That Have the Potential to Utilize BDE-209 as a Sole Carbon Source

The bacterial strains isolated from the samples taken on days 43, 105 and 140 during the biodegradation in the clay/water slurry reactor and their ability to utilize the BDE-209 as a sole carbon source are shown in Table 1. The phylogenetic tree of these isolated and identified bacteria is shown in Figure S1, where eight bacterial strains were identified. These consisted of three *Acinetobacter* sp. strains which are members of the γ-proteobacteria (NH-1, NH-7 and NH-8), three *Achromobacter* sp. strains which are members of the β-proteobacteria (NH-2, NH-4 and NH-6), one *Pseudomonas* sp. strain which is a γ-proteobacteria (NH-5) and one *Bacillus* sp. strain which is a member of the Firmicutes (NH-3). Using the Biolog MT2 microplates method, *Achromobacter* sp. NH-2 was characterized as being able to strongly utilize BDE-209 as a sole carbon source; the comparative abilities to utilize BDE-209 as a sole carbon source were medium in four of the above mentioned strains (NH-1, NH-4, NH-6 and NH-7) and weak in the three additional strains (NH-3, NH-5 and NH-8). Four bacterial strains (NH-1, NH-2, NH-3 and NH-4) were isolated on day 43 after the pretreatment process involving the photolysis acted as an enhancing factor in terms of the biodegradation process. These strains were able to utilize the metabolites created by the photolytic process, namely the low bromides PBDEs and used these for biological co-metabolism with BDE-209 in the SSR.

## 4. Discussion

### 4.1. A Comparison of BDE-209 Degradation in Sorbent/Water System

UVB irradiation was found to be an effective process for degrading BDE-209 in the clay/water slurry system yielding a first order rate constant *k* = 0.017 1/day during 35 days of treatment. In contrast, the UVB-based photolysis process was unable to successfully degrade BDE-209 in the soil/water slurry system (*k* = 0 1/day). A prior study showed similar differences in the degradation of BDE-209 associated with clays vs. soils. Specifically, in a slurry reactor with continuous UV irradiation (300–400 nm) for 14 days the results were as follows: Na+-montmorillonite (a 2:1 clay mineral) (*k* = 0.0192 1/day) > kaolinite (a 1:1 clay mineral) (*k* = 0.0158 1/day) > loam sediment with 16.4% organic carbon (*k* = 0.0046 1/day) [13]. Na+-montmorillonite has a stronger affinity for BDE-209 than other minerals and sediments. In this study, the photolytic degradation of BDE-209 (*k* = 0.017 1/day) by UVB irradiation in the clay-water slurry was slower than that in the Na+-montmorillonite, which was likely due to the differences in the experimental setup and conditions, such as the process in a saturated water environment or the 312nm-UVB irradiation in the Ca-montmorillonite/water system. Another study found that the percentage of BDE-209 remaining after the treatment of various geosorbents with UV irradiation (300–400 nm) for 64 h were: lake sediment (57%) > sand (21%) > silica gel (1%), and in two samples the significant levels of BDE-209 remained after 244 h, i.e., a Nordic reference soil (38%) and the lake sediment (26%) [6]. A possible reason for the varying efficiency of BDE-209 photolysis in the different sorbents is their composition, specifically the UV shielding effects and UV sorption of various particles comprised in each sorbent. In the present study, the montmorillonite clay should manifest comparatively low UV shielding and more scattering of the radiation. The greater scattering by the clay particles should increase the fluence rate of the reactor near the UV lamp [14]. In the case of the whole soil, the sorption of UV radiation, especially by the natural organic matter present, would be expected to reduce the fluence rate and to affect the half-lives of the free radicals. Humic acids (HA) and fulvic acids in natural soils act as photon traps and hence could provide an optical (UV) filter effect thereby decreasing the photodegradation rate of BDE-209 and similar POPs [15]. A previous study has shown that BDE-209 photodegradation during 72 h of solar irradiation was 29.6% in an aqueous HA solution, as compared to 70.8% in pure water [7]. Likewise, the sorption of BDE-209 to HA-coated sand particles (0.071% total organic carbon) reduced the photolytic degradation during a 96-h exposure to solar irradiation from 70.8% (in pure water) to 10.9%.

The two different sorbents used (clay vs. soil) have been previously shown to affect the rate of BDE-209 biodegradation. In the clay/water slurries, the BDE-209 biodegradation displayed first order kinetics with a rate constant of 0.026 1/day (Figure S2), almost four times faster than that in the soil/water slurries (0.0066 1/day) [10]. In the present study, BDE-209 biodegradation was plausibly slower in a soil/water slurry system due to the sorption of BDE-209 by the soil organic matter (SOM) which is widely thought to function as a partition phase for poorly water-soluble nonionic organic contaminants (NOCs) [16]. BDE-209 has a relatively high Kow (Log Kow = 6.65) which manifests a high partition coefficient (Kom) into the SOM phase. This resulted in a significant amount of BDE-209 being partitioned into the SOM with relatively less mass dissolved in the aqueous phase where it was maximally bioavailable [17]. The sorption of hydrophobic organic compounds by the clay mineral micropores (<2 nm) and hydrophobic domains between the changeable cations [18] could plausibly play a key role in controlling NOC fate and transport in porous particles with a very low level of SOM (<0.1%) [19]. The adsorption of NOCs in such hydrophobic domains is limited in the presence of water which hydrates exchangeable cations and obscures much of the clay mineral surface area [18]. Thus, in this study, BDE-209 might have been minimally adsorbed onto the surface of montmorillonite clay due to the presence of water, which hydrated exchangeable cations and occupied most of clay mineral surface. The biodegradation of BDE-209 was likely facilitated by the fact that a greater portion was dissolved in the aqueous phase of the clay/water slurries, in contrast to the soil/water slurries where most of the BDE-209 was partitioned into the SOM.

### 4.2. Influence of UV Irradiation on BDE-209 Degradation in a Clay/Water System

A primary mechanism of the PBDE photodegradation includes the consecutive reductive debromination and intramolecular elimination of HBr in soils [5]. The possible intermediates that were measured included lower brominated products, hydroxylated BDEs, bromophenols, brominated and hydroxylated dibenzofurans (PBDFs) and dioxins (PBDDs). An accelerated degradation might be explained by the H-donor capacity of water which can promote debromination as well as hydroxylation [20]. Thus, a significant number of electrons was required for the sequential reductive reactions of BDE-209 by photolysis in a sorbent/water system. For example, the total concentrations of 23 PBDEs congeners were significantly increased after 35 days by the photolytic process (Figure S3). The highly brominated PBDEs (Octa-BDEs and Nona-BDEs) increased by almost two-fold from 1260.05 ng/g on day 0 to 2741.01 ng/g on day 35, while the PBDEs with low levels of bromination (Tri-BDEs, Tetra-BDEs, Penta-BDEs, Hexa-BDEs, and Hepta-BDEs) increased by about 1.5 fold from 83.13 ng/g to 119.67 ng/g.

The photolysis rate of BDE-209 is influenced by the wavelength and intensity of UV irradiation, which can affect the quantum yield [13,17]. UV wavelength is a more important factor than UV intensity, although increasing the UV intensity enhances the possibility of contact between the BDE-209 and the photons in the bioreactor. The photon energy cannot be changed without altering the UV wavelength. A previous study indicated that the activating wavelength for the radical formation from BDE-209 was in the range of UVA (400–320 nm) to UVB (320–280 nm) [21]. High relative radical yields were achieved using a xenon arc lamp for irradiation on BDE-209 (14 m) with a shorter 280 nm cutoff filter (black bars) as compared to that achieved with a 309 nm cutoff filter. Similar trends were observed for the triclosan photodegradation efficiencies, i.e., 90% to 98% and 79% to 90% at the wavelengths of 254 and 365 nm, respectively [22]. In this study, the absorption spectrum of BDE-209 by the full scan function of 200–400 nm UV/VIS spectrophotometer (Optizen^®^ 2120 UV plus, Mecasys) was identified at 312 nm or 365 nm UV irradiation. The absorption spectrum of BDE-209 itself and in clay/water slurry systems are shown in Figure S4. Although the absorbance of BDE-209 was not the best in the range of 200–400 nm, the absorbance itself was measured as 0.206 at 312 nm and 0.005 at 365nm. The absorbance of BDE-209 in the clay/water system was measured as 0.206 at 312 nm and 0.355 at 365 nm. Besides, UVC irradiation theoretically performs a better removal of BDE-209 by photolysis based on previous research [5]. However, the continuous UVC irradiation of soil/clay-slurries at the energy intensity used in this study will result in increased energy consumption, which will increase significantly the treatment cost of *ad-situ* or *ex-situ* remediation. In addition, UVC light with a shorter-wave (280–200 nm) in practice damages human DNA and creates an increased risk of skin cancer. Indigenous microorganisms in the soil are unable to survive during UVC irradiation. This study selected UVB irradiation to reduce the health risks to staff associated with UVC irradiation exposure during the SSR operation.

Two other environmental factors pertinent to the reactor system used here might provide electrons in addition to those produced by the UVB irradiation itself. Firstly, clay minerals are known to have a significant electron-donating ability during debromination [13]. The montmorillonite clay used is a member of the smectite group; these clays are 2:1 phyllosilicate mineral, consisting of an Al sheet between two Si sheets arranged in a tetrahedral–octahedral– tetrahedral structure. The bridging oxygen of the Si–O–Al structure within montmorillonite has a high electron density [13] and it is likely that some of the electrons donated during UVB photolysis and utilized for BDE-209 debromination were derived from these bridging oxygens present in the montmorillonite. Secondly, water content is likely to play an important role in PBDEs photodegradation on solid matrices because water itself is adsorbed to the surfaces of geosorbent particles in the soil via hydrogen bonding and dipole interactions [5]. Water molecules in such sorbent/water systems are able to act as hydrogen donors and this could enhance the photolytic reaction of BDE-209. For example, the half-life of BDE-209 during the UVB photolysis in the clay/water slurry system was 33 days in this study. Previously, in systems containing an unsaturated water content (2 mg/μL) it was found to be less than 36 days using montmorillonite or 44 days using kaolinite [13].

Pretreatment by 35-day UV irradiation appeared to influence the BDE-209 biodegradation in the clay/water slurry system used herein. This UV photolysis pretreatment appeared to increase the rate of BDE-209 biodegradation in the clay/water slurry system by allowing co-metabolism during the subsequent biodegradation step when compared to systems without prior UV treatment. The first-order-rate constant from this study (with UV pretreatment) was 0.026 1/day, which is almost 2.8-fold higher (0.0092 1/day) than that obtained for biodegradation under the same incubation conditions and using the same bacterial inoculation (obtained from Nei-Hu WWTP) but lacking UV pretreatment [9]. The concentration of PBDEs considered to be co-metabolites was largely decreased during biodegradation. The concentrations of PBDEs with low levels of bromination, mostly Hepta-BDEs (BDE-183, BDE-184 and BDE-191) and Hexa-BDEs (BDE-138, BDE-153 and BDE-154) were found to have decreased from 73.24 ng/g (day 43) to 21.05 ng/g (day 140). Similarly, the concentrations of Tri-BDE (BDE-17) and Tetra-BDE (BDE-47) were found to be decreased from the 74th day to the 140th day. Previous studies have shown the accelerated biodegradation of high-molecular-weight PAHs such as anthracene, pyrene, benz[a]anthracene and dibenz-[a,h]anthracene following UV pretreatment (254 nm UVC irradiation at a light intensity of 1.68 × 10^−8^ Einstein/L.s for 3–6 h) [23].

### 4.3. Bacterial Strains Isolated Involved in the BDE-209 Biodegradation in a Clay/Water System

Eight bacterial strains capable of degrading PBDEs or their biodegradation products were isolated from the clay/water slurry system. These strains included *Achromobacter* spp., *Acinetobacter* spp. and a *Bacillus* sp., which were able to utilize BDE-209 as a sole carbon source. *Achromobacter* are Gram-negative β-proteobacteria. They are strictly aerobic and commonly found in water and soils. One example, *Achromobacter piechaudii*, isolated from contaminated desert soil, was able to biodegrade 2,4,6-tribromophenol and produce bromide under aerobic conditions [24]. In addition, *Achrormobacter* can use specific polychlorinated biphenyls (PCB) congeners as a sole carbon and energy source for growth; these chemicals have structural similarity to PBDE congeners [25]. Three *Acinetobacter* strains (NH-2, NH-4, NH-6) were also isolated; these are γ-proteobacteria and were confirmed to biodegrade PBDEs. *Acinetobacter* have been reported to grow on agar containing 0.5 mg/L BDE-209 and hence are considered as BDE-209-degrading bacteria; these strains were isolated from river sediments [26]. A mixed bacterial community obtained from BDE-209-contaminated soils contained *Acinetobacter* sp. able to carry out BDE-100 biodegradation [27]. In another microcosm study, under anaerobic conditions, the *Acinetobacter* spp. present in a mixed culture (containing 10 µM BDE-209, 20 g wet weight sediments and 150 mL MSB) increased when alternative electron donors such as methanol and ethanol were provided [28]. In the present study, a single Gram-positive *Bacillus* sp. strain capable of BDE-209 biodegradation was identified. A previous study showed that the metal-resistant bacterial strain *Bacillus cereus* JP12 was able to use BDE-209 as a sole carbon and energy source when grown on MSB. Using this strain, more than 88% of the BDE-209 present (1 mg/L) was biodegraded over 12 days at pH 6.0 and 30 °C [29]. A single strain of *Pseudomonas* with a medium ability to utilize BDE-209 was isolated during our study on day 105.

### 4.4. The Bacterial Communities Involved in the BDE-209 Biodegradation in a Clay/Water System

Many bacteria identified in this study were capable of biodegrading BDE-209 as well as other PBDE congeners and aromatic POPs. For example, the percentage of *Acidovorax* spp., which is a member of the β-proteobacteria, present in the total bacterial population, was found to be the highest at 58.88% on day 43 in our clay/water slurry system; this was calculated as a relative abundance against the total community. *Acidovorax* is Gram-negative and a member of the β-proteobacteria; this species is known to be able to degrade many POPs/EDCs, especially aromatic compounds in oligotrophic groundwater [30]. The SOM content in the sorbent/water system is a critical factor and can have a strong influence on the microbial biomass and its community structure. *Acidovorax* spp. have been identified in the high-level BDE-209-contaminated microcosm containing lower levels of SOM soil [31]. In this study, *Acidovorax* spp. in the lower-SOM clay/water system was able to utilize BDE-209 (7.72 ± 2.01 μg/L) as a single carbon source by generating target enzymes such as those encoded by the Phn genes and Bph genes, both of which are capable of cleaving the structure of diphenyl ether [32,33]. When more metabolites e.g., PBDEs and hydroxylated PBDEs are present in the clay/water slurry system, the dominant bacterial groups present in the microcosms are changed and seem to prefer to utilize these molecules as carbon sources. The percentage of *Acidovorax* spp. can therefore be seen to decrease to 17.89% on day 105 and 2.09% on day 140 during biodegradation. On the other hand, *Sediminibacterium* spp., present in the original bacterial mixed culture inoculum was dominant during the second stage of BDE-209 biodegradation and was then replaced by other bacterial species. The percentage of *Sediminibacterium* spp. present during BDE-209 biodegradation in the samples collected from the clay/water slurries gradually decreased from 8.67% on day 43, to 2.32% on day 104 and then to 1.96% on day 140; this was calculated as a relative abundance against the total community. *Sediminibacterium* spp. are aerobic bacteria often found in aquatic sediment, activated sludge, surface water and waste reservoirs under solar irradiation. A relatively high percentage (11.76%) of *S. ginsengisoli* was identified in the microcosms under continuous exposure to 365-nm UV irradiation at 1.5~2.0 mw/cm^2^ in the presence of a high level of BDE-209, compared to their absence in the dark environment [31].

Most of the bacteria identified as present in the clay/water slurries bacterial community were dominant on day 105, indicating that they played an important role in BDE-209 degradation and possibly the biodegradation of other metabolites. For example, the highest percentage of *Alicycliphilus* spp. detected during BDE-209 biodegradation was 20.37% on day 105, which should be compared to 0.52% on day 43 and 1.13% on day 140; this was calculated as a relative abundance against the total community. *Alicycliphilus* spp. are facultative Gram-negative bacteria commonly found in activated sludge from WWTP, waterbodies and sediments, and are known to degrade a wide variety of aromatic compounds. *Alicycliphilus* spp. have been found to degrade pentachlorophenol in aerobic granular sludge and they have also been shown to be responsible for the breakdown of a phenol during the activated-sludge process [34]. *A. denitrificans* K601 has been reported to be able to biodegrade BDE-209 [35]. Moreover, *Pseudomonas* spp. are facultative Gram-negative bacteria found during the course of BDE-209 biodegradation in all the collected samples; they made up 5.12% of the total bacterial population on day 43, were dominant at 12.07% on day 105 and were still quite dominant at 6.08% on day 140, calculated as a relative abundance against the total community. *Pseudomonas* spp. have been shown to metabolize benzene and other aromatic compounds [30]. *P. aeruginosa*, when added to PBDE-contaminated soil, was found to biodegrade BDE-209 [36]. BDE-209 debromination occurred sequentially resulting in various debrominated PBDE products including nona-BDEs (BDE-208, and BDE-207), octa-BDE (BDE-203, BDE-202, BDE-197 and BDE-196), and hepta-BDEs (BDE-183) [37]. *P. dioxanivorans* CB1190 have also been shown to biodegrade lower brominated PBDEs [38]. In addition, the *Novosphingobium* spp. present were α-Proteobacteria Gram-negative aerobic bacteria which are commonly found in waterbodies, soil and sediment. The percentages of *Novosphingobium* spp. present during BDE-209 biodegradation in the clay/water slurries were 6.56% on day 43, a dominant 6.72% on day 105 and 4.73% on day 140; this was calculated as a relative abundance against the total community. *Novosphingobium* spp. have been shown to degrade many POPs/EDCs including chlorophenols and PAHs [39,40]. *N. chloroacetimidivorans* isolated from activated sludge have been shown to biodegrade pesticides including butachlor, acetochlor and alachlor [41]. Furthermore, the dominant percentage of *Ramibacter* spp. detected during BDE-209 biodegradation reached 7.13% on day 105. *Ramibacter* spp. are β-proteobacteria Gram-negative aerobic bacteria that have been found in desert sands. *R. tataouinensis* has been reported to possess significant DNA repair mechanisms in the form of highly active superoxide dismutase that is able to remove reactive oxygen species allowing the bacterium to tolerate oxidative stress. It is inferred that *Ramibacter* spp. might be irrelevant to the biodegradation of BDE-209 and its metabolites, but these bacteria may be able to survive well in the clay/water slurries when there is continuous UV irradiation.

The percentage of *Desulfomonile* spp. was 43.21% on day 140 of BDE-209 biodegradation. *Desulfomonile* spp. are Gram-negative strictly anaerobic bacteria often found in activated sludge from WWTP and in river sediments. This dominant species is likely to be present in a range of different anaerobic micro-environments within the clay/water slurry system where there are higher levels of electron acceptors such as PBDEs. Chlorophenol and trichlorophenol are reductively dechlorinated by *Desulfomonile tiedjei* DCB-1 [42]. It can be inferred from the above findings that biological debromination was occurring in the clay/water system on day 140. Moreover, *Sphingomonas* spp. were identified on day 105 (3.04%) and day 140 (dominant at 7.00%) in the clay/water slurry system; this was calculated as a relative abundance against the total community, possibly because they were unitizing the low-brominated PBDEs that were present. *Sphingomonas* sp. are Gram-negative facultative anaerobes that have been shown to have the ability to biodegrade low-brominated PBDEs. *Sphingomonas* sp. SS3 has been shown to biodegrade 4-bromodiphenyl ether [38]. *Sphingomonas* sp. PH-07 is capable of biodegrading PBDE congeners such as 4-Bromodiphenyl ether, 2,4-dibromodiphenyl ether and BDE-15 when they are present in a diphenyl ether solution [43].

## 5. Conclusions

A sequential UV photolysis-microbial degradation process was developed and characterized which effectively removed BDE-209 in a clay/water slurry system. The pure clay mineral used (montmorillonite), along with the water molecules present, are likely to have provided sufficient electrons for a series of photolytic reactions. The dominant bacterial communities identified have been shown in prior studies to have the capacity for PBDE biodegradation. The relative abundances of major genera at the community level were found to have changed significantly at the different stages of biodegradation. *Acidovorax* spp. and *Sediminibacterium* spp. were dominant during the initial period (day 43) during which there was the biodegradation of the high-brominated PBDEs (e.g., BDE-209). *Alicycliphilus* spp., *Acidovorax* spp., and *Pseudomonas* spp. were dominant during the middle period (day 105), which involved the utilization of various metabolites such as the low-brominated PBDEs and hydroxylated PBDEs (OH-PBDEs). *Desulfomonile* spp. were dominant during the last period (day 140), which suggested that biological debromination was occurring in clay-water slurry microenvironments. Various bacterial strains such as *Achromobacter* spp., that were isolated from the sequential UV photolysis-microbial degradation process, were found to have the ability to utilize BDE-209 as sole carbon source. These findings provide the basis of a novel approach for degrading BDE-209 and similar ECs. More detailed experiments are required to further optimize the design and functioning of a practical *ad-situ* or *ex-situ* system for the remediation of POP/EDC-contaminated solids using a slurry bioreactor.

## Figures and Tables

**Table 1:** Isolated bacterial strains identified during the biodegradation and their ability to utilize BDE-209 as a sole carbon source by the Biolog MT2 microplate method.

No.	Biodegradation Sampling Day	Bacterial Species (Similarity)	Response to BDE-209 Utilization
NH-1	43	*Acinetobacter* sp. (99%)	2
NH-2		*Achromobacter* sp. (99%)	3
NH-3		*Bacillus* sp. (99%)	1
NH-4		*Achromobacter* sp. (99%)	2
NH-5	105	*Pseudomonas* sp. (99%)	1
NH-6		*Achromobacter* sp. (99%)	2
NH-7	140	*Acinetobacter* sp. (99%)	2
NH-8		*Acinetobacter* sp. (99%)	1
**Level**	**1:Weak**	**2:Medium**	**3:Strong**
OD_590_	<0.1	0.1–0.3	>0.3

## Supplementary Materials

The following are available online at https://www.mdpi.com/2076-2607/8/5/633/s1, Figure S1: Phylogenetic analysis of BDE-209-utilizing isolates present during biodegradation in a clay/water slurry system and various other related species based on their 16S rRNA gene sequences, Figure S2: A comparison between a sequential UVB photolysis-biodegradation process (● and ○) in the clay/water slurries and biodegradation (▉) in the soil/water slurries in an absolutely dark environment carried out using the Nei-Hu bacterial community, Figure S3: The concentrations of 23 PBDE congeners after UVB photolysis in the clay/water system: (a) Octa-BDEs and Nona-BDEs; (b) Hepta-BDEs, Hexa-BDEs, Tri-BDEs, Tetra-BDEs, and Penta-BDEs. Ori is defined as the original clay; day 0 is defined as the clay/water system after BDE-209 addition, Figure S4: The 200–400nm UV transmittance spectrum of (a) BDE-209 itself (blue line) and (b) aqueous solution in the clay/water slurry system (red line). Orange arrows are the wavelength of 312 nm and 365 nm, respectively, Table S1: Studies that have used a UV-biological sequential treatment process to treat POPs/EDCs, Table S2: Chemical-physical characteristics of sorbents used in this study, Table S3: The number of sequences on day 43 sample generated by Illumina 454 sequencing platform, Table S4: The number of sequences on day 105 sample generated by Illumina 454 sequencing platform, Table S5: The number of sequences on day 140 sample generated by Illumina 454 sequencing platform.
