# Supplementary material for: Characterization of a Sequential UV Photolysis-Biodegradation Process for Treatment of Decabrominated Diphenyl Ethers in Sorbent/Water Systems
Source: Microorganisms. 2020 Apr 27;8(5):633. doi: 10.3390/microorganisms8050633 (PMC7284435; doi:10.3390/microorganisms8050633)
Supplement: Supplementary file 1 [file microorganisms-08-00633-s001.pdf]

microorganisms

Supplementary Data

Characterization of a sequential UV Photolysis-Biodegradation Process for Treatment of Decabrominated Diphenyl Ethers in Sorbent/Water Systems

Yi-Tang Chang [1,2,*], Wei-Liang Chao [1], Hsin-Yu Chen [1], Hui Li [2] and Stephen A. Boyd [2,*]

[1] Department of Microbiology, Soochow University, Shilin District, Taipei, 11102, Taiwan; wlchao@scu.edu.tw (W.-L.C.); fish3265175@gmail.com (H.-Y.C.)

[2] Department of Plant, Soil and Microbial Science, Michigan State University, East Lansing, 48824, USA; lihui@msu.edu

* Correspondence: ytchang@scu.edu.tw(Y.-T.C.); boyds@msu.edu (S.B.); Tel.: +886-2-7369277 EXT 6862 (Y.-T.C.); +1-517-881-0579 (S.B.)

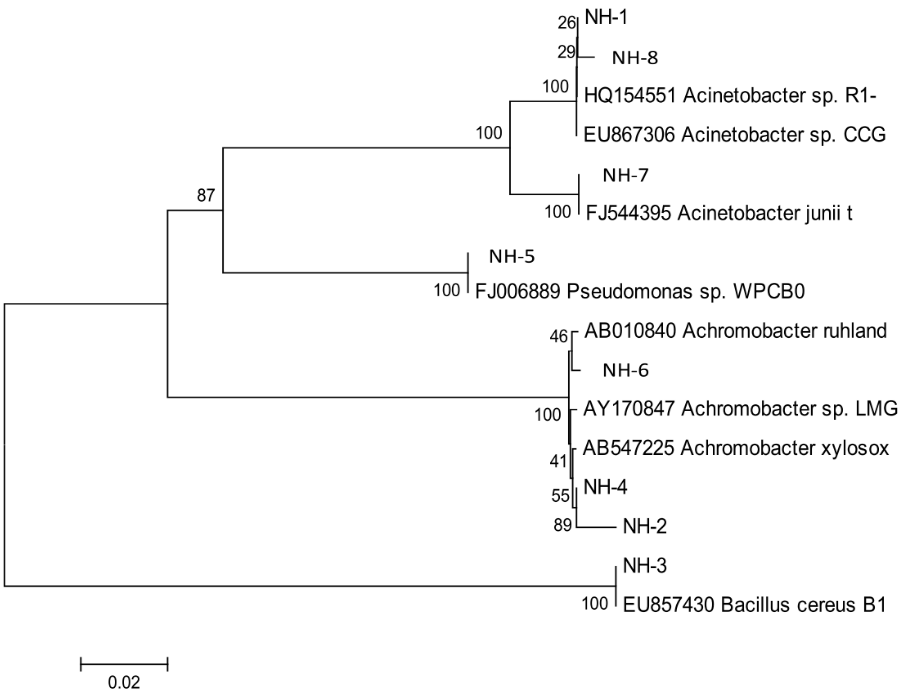

Figure S1. Phylogenetic analysis of BDE-209-utilizing isolates present during biodegradation in a clay/water slurry system and various other related species based on their 16S rRNA gene sequences [1].

[1]: The phylogenetic tree was created using the neighbor-joining method with bootstrapping. The values indicated at the branch points are based on 100 bootstrap replications (%). The *scale bar* represents 0.5 substitutions per nucleotide position.

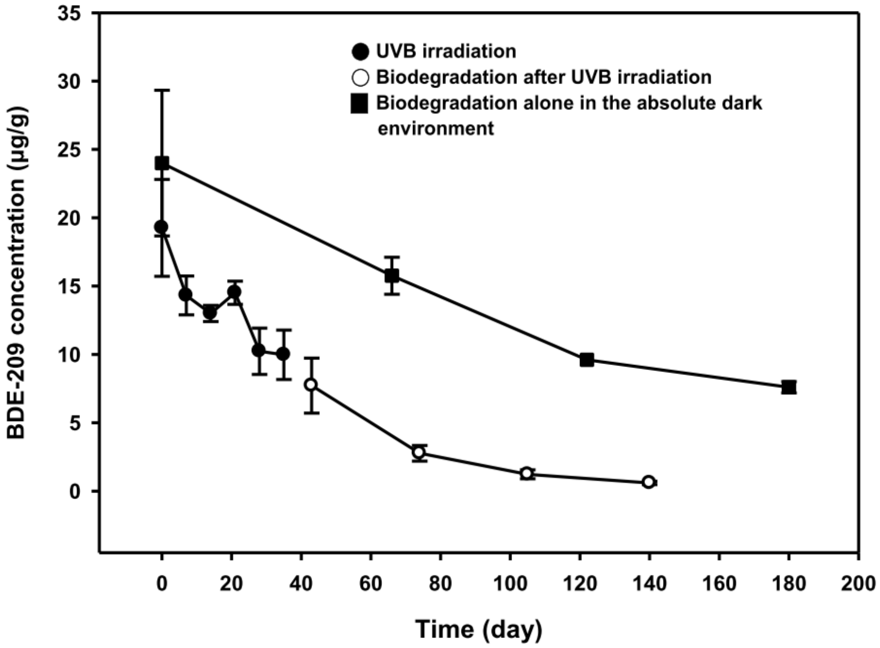

Figure S2. A comparison between a sequential UVB photolysis-biodegradation process (●and○) in the clay/water slurries and biodegradation (■) in the soil/water slurries in an absolute dark environment carried out using the same Nei-Hu bacterial community.

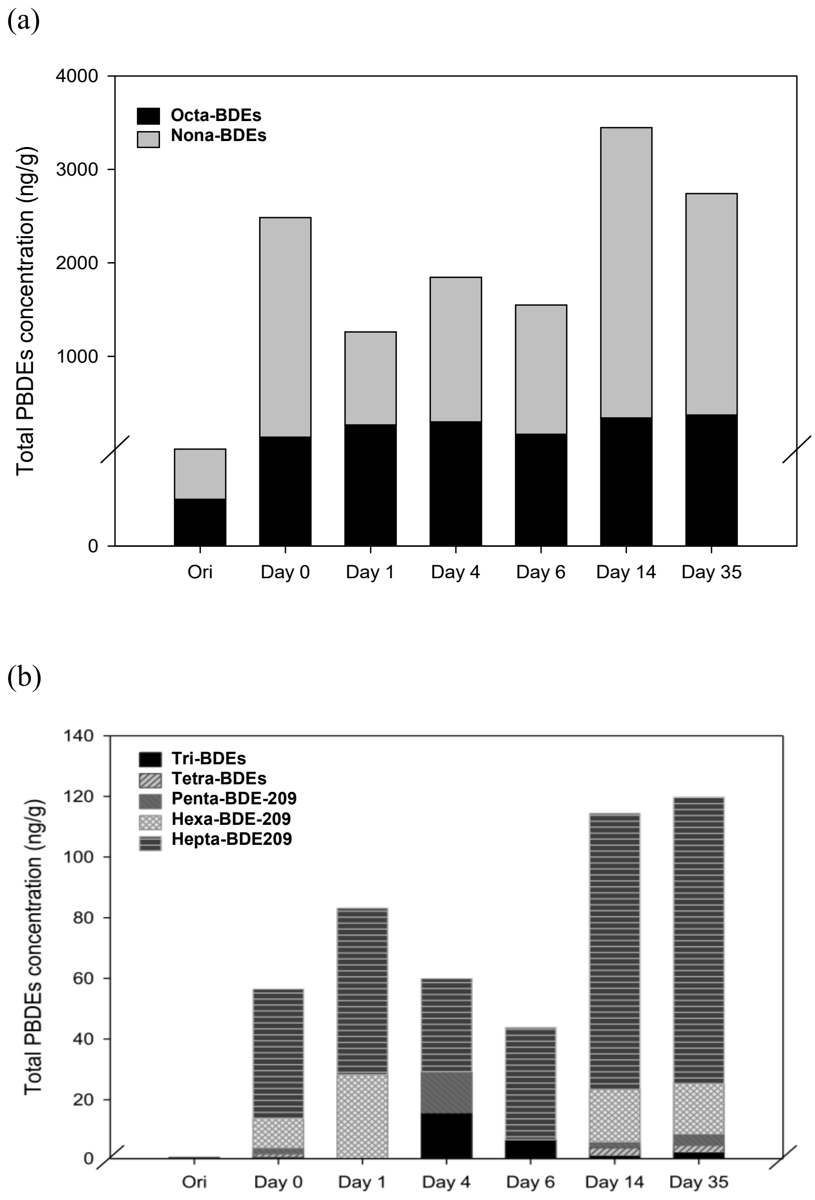

Figure S3. The concentrations of 23 PBDE congeners after UVB photolysis in the clay/water system[1]: (a) Octa-BDEs and Nona-BDEs; (b) Hepta-BDEs, Hexa-BDEs, Tri-BDEs, Tetra-BDEs, and Penta-BDEs. Ori is defined as the original clay; Day 0 is defined as the clay/water system after BDE-209 addition.

[1]: The 23 PBDE congeners included BDE-17, -28, -47, -49, -66, -71, -77, -85, -99, -100, -119, -126, -138, -153, -154, -156, -183, -184, -191, -196, -197, -206, and -207.

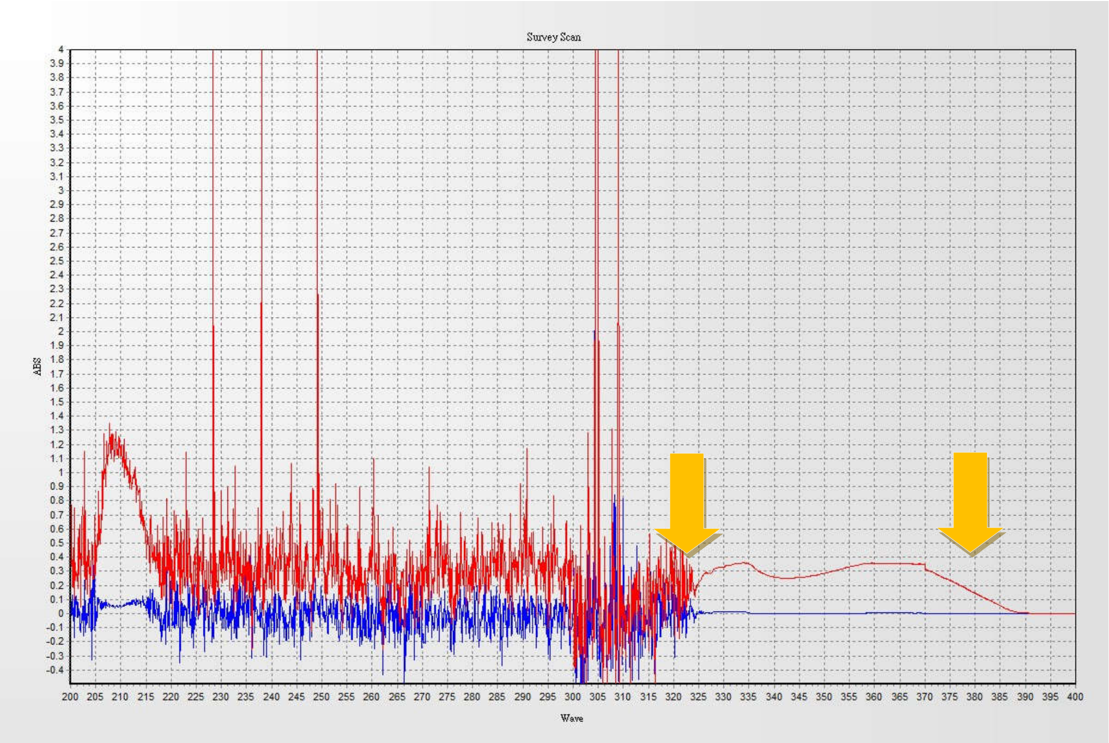

Figure S4. The 200-400nm UV transmittance spectrum of (a) BDE-209 itself (blue line) and (b) aqueous solution in the clay/water slurry system (red line). Orange arrows are the wavelength of 312 nm and 365 nm, respectively.

Table S1. Studies that have used a UV-biological sequential treatment process to treat POPs/EDCs.

Media	Target compounds	UV lamp and light intensity	Bacterial species	Best removal of POPs/EDCs	References
Liquid: aqueous solution	Isoproturon	36 W black actinic light (330-390 nm) with immobilized TiO_2 supported on glass rings	Mixed culture obtained from activated sludge of municipal WWTP	Isoproturon: 100% Dissolved organic carbon: 95%	[1]
Liquid: soil extracts	Mixed Polycyclic aromatic hydrocarbons (3, 4 and 5 rings)	18W UV blue lamps (30% UV-A–5% UV-B) and 0.25 mW/cm	Single strain: *Pseudomonas* sp.	Fluorene: 94% Phenanthrene: 100% Anthracene 100% Fuoranthrene 14% Pyrene 46% Benzo(a)anthracene 100% Benzo(a)pyrene 100%	[2]
Liquid: mineral salt medium (MSM)	A mixture of chlorophenols: 4-chlorophenol (4-CP), 2,4-dichlorophenol (DCP), 2,4,6-trichlorophenol (TCP), and pentachlorophenol (PCP)	18W UV blue-lamps (30% UVA–5% UVB) and 300 $\mu W/cm^2$	Activated sludge of WWTP mixed culture	4-CP: 100% DCP: 100% TCP: 100% PCP: 100%	[3]
Liquid: phosphate buffer	Chlorophenols: 2-Chlorophenol (2-CP), DCP, TCP and PCP	UV/TiO_2 (anatase form)	Single strain: *Trametes pubescens*	With glucose, removal of 100% of chlorphenols. Without glucose: 2-CP:82.8% DCP:91.1% TCP:79.3% PCP:94.5%	[4]
Liquid: artificial seawater media	Dibenzothiophene (DBT)	254 nm UV at 2,000 mJ/cm^2	Mixed microbial culture enriched by DBT	81%	[5]
Liquid: mineral salt medium	2, 4-dichlorophenoxyacetic acid (2,4-D)	256 nm UV/TiO_2	Mixed culture consisting of an active isolated consortium enriched by 2,4-D	100%	[6]
Soil Slurry (sorbent/water systems)	Decabromodiphenyl ether (BDE-209)	312 nm UVB at 0.58-0.97 mW/cm^2	Mixed culture consisting of bacteria able to biodegrade BDE-209	96.88%	This study

References

1. Parra, S.; Malato, S.; Pulgarin, C. New integrated photocatalytic-biological flow system using supported TiO_2 and fixed bacteria for the mineralization of isoproturon. *Appl. Catal. B* **2002**, *36*, 131-144.
2. Guieysse, B.; Viklund, G. Sequential UV-biological degradation of polycyclic aromatic hydrocarbons in two-phases partitioning bioreactors. *Chemosphere* **2005,** *59*, 369-376.
3. Tamer, E.; Hamid, Z.; Aly, A.M.; Ossama E.T.; Bo, M.; Benoit, G. Sequential UV-biological degradation of chlorophenols. *Chemosphere* **2006,** *63*, 277-284.
4. González, L.F.; Sarria, V.; Sánchez, O.F. Degradation of chlorophenols by sequential biological-advanced oxidative process using *Trametes pubescens* and TiO_2/UV. *Bioresour. Technol.* **2010,** *101*, 3493-3499.
5. Cooper, E.M.; Stapleton, H.M.; Matson, C.W.; Di Giulio, R.T.; Schuler, A.J. Ultraviolet treatment and biodegradation of dibenzothiophene: identification and toxicity of products. *Environ. Toxicol. Chem.* **2010,** *29*, 2409-2416.
6. Samir R., Essam T., Ragab Y., Hashem A., Enhanced photocatalytic-biological degradation of 2, 4-dichlorophenoxyacetic acid. *Bull. Fac. Pharm. Cairo Univ.* **2015,** *53*, 77-82.

Table S2. Chemical-physical characteristics of sorbents used in this study[1].

Soil	Composition (%)			Source	BET-(N_2) SA(m^2/g)	SOM (%)	CEC (meq/ 100g)
	Sand	Slit	Clay				
**Clay**	-	-	> 99	Gonzales County, TX, USA	80.79	≒0	120.2
Soil	10	54	36	Taichung, Taiwan	2.10	1.883	1.37

[1]: Measured by the Soil Survey and Testing Center, National Chung-Hsing University, Taichung, Taiwan.

Table S3 The number of sequences on Day 43 sample generated by Illumina 454 sequencing platform

Taxonomic Level	Reads Classified to Taxonomic Level [1]	Total Reads Classified to Taxonomic Level (Coverage %)	Total Taxonomic Level Categories Identified
Kingdom	324,179	100.00	1
Phylum	323,780	99.88	14
Class	321,904	99.30	25
Order	320,823	98.98	55
Family	318,745	98.32	106
Genus	309,856	95.58	245

[1]: The "Other" category is the sum of all classifications with less than 3.50 % abundance.

Table S4. The number of sequences on Day 105 sample generated by Illumina 454 sequencing platform.

Taxonomic Level	Reads Classified to Taxonomic Level [1]	Total Reads Classified to Taxonomic Level (Coverage %)	Total Taxonomic Level Categories Identified
Kingdom	321,311	100	1
Phylum	319,799	99.53	17
Class	316,517	98.51	36
Order	312,024	97.11	71
Family	309,022	96.17	141
Genus	295,147	91.86	304

[1]: The "Other" category is the sum of all classifications with less than 3.50 % abundance.

Table S5. The number of sequences on Day 140 sample generated by Illumina 454 sequencing platform.

Taxonomic Level	Reads Classified to Taxonomic Level [1]	Total Reads Classified to Taxonomic Level (Coverage %)	Total Taxonomic Level Categories Identified
Kingdom	352,050	100.00	1
Phylum	337,900	95.98	21
Class	332,808	94.53	41
Order	323,556	91.90	81
Family	315,265	89.55	165
Genus	305,110	86.66	359

[1]: The "Other" category is the sum of all classifications with less than 3.50 % abundance.
